# Identification of focal ARDS using PF ratio: a cross-sectional study

**DOI:** 10.1186/s13054-020-03190-0

**Published:** 2020-07-29

**Authors:** Kay Choong See, Juliet Sahagun, Juvel Taculod

**Affiliations:** 1 Division of Respiratory & Critical Care Medicine, Department of Medicine, National University Hospital, 1E Kent Ridge Road, NUHS Tower Block Level 10, Singapore, 119228 Singapore; 2 Department of Medicine, Yong Loo Lin School of Medicine, National University of Singapore, Singapore, Singapore; 3 Division of Critical Care – Respiratory Therapy, National University Hospital, Singapore, Singapore

**Keywords:** Diagnosis, Lung, Respiratory distress syndrome, Adult, Ultrasonography

In normal lungs, ventilation exceeds perfusion in lung areas above the heart while perfusion exceeds ventilation in lung areas below the heart (i.e., in the posterobasal lung areas when a person is supine) [1]. When the lung bases are diseased, perfusion exceeds ventilation more markedly, gas exchange within the affected alveoli gets diminished, the partial pressure of alveolar oxygen falls, and hypoxemia ensues. To mitigate this mismatch of perfusion and ventilation, hypoxic vasoconstriction occurs in areas of poorly ventilated lung.

However, hypoxic vasoconstriction is impaired in acute respiratory distress syndrome (ARDS) [2, 3]. As such, if consolidation exists predominantly in the posterobasal lung regions (i.e., focal ARDS) [4], we can expect that greater ventilation-perfusion mismatch could reduce oxygenation more severely, as measured using the partial pressure of arterial oxygen divided by fraction of inspired oxygen (PF ratio). If an association between focal ARDS and PF ratio can be shown, PF ratio could then be used in place of thoracic imaging to identify focal ARDS. We therefore hypothesized that focal ARDS is associated with lower PF ratio and investigated this relationship.

We included patients with ARDS fulfilling the Berlin Definition, who were admitted to our Medical Intensive Care Unit in 2014–2017, and who only received invasive mechanical ventilation. On admission, trained respiratory therapists performed a 12-point lung ultrasound using a 2–4 MHz phased array transducer and semi-quantitatively scored each region [5]. We identified focal ARDS on lung ultrasound [6], if the consolidated regions were only present in the posterobasal regions (combination of lower lateral, upper posterior, and lower posterior regions) and absent in the anteroapical regions (combination of upper anterior, lower anterior, and upper lateral regions) [4].

The association of focal ARDS with PF ratio was analyzed using logistic regression and with PF ratio (taken at the time of the lung ultrasound scan) as a continuous variable. To check for any nonlinear relationship of focal ARDS with PF ratio, we fitted a logistic regression model using a restricted cubic spline with four knots and taking the PF ratio of the first knot as the reference level. Wald test for linearity was then done (*P* < 0.05 indicates non-linearity).

One hundred fifty-two patients were studied (age 63.3 ± 14.1 years; 53 (34.9%) female; ICU mortality 16.5%; hospital mortality 33.6%). Admission diagnoses were as follows: pneumonia (61 patients; 40.1%), non-pneumonia sepsis (19; 12.5%), chronic obstructive pulmonary disease (9; 5.9%), acute myocardial infarction (3; 2.0%), stroke (12; 7.9%), and other diagnoses such as massive hemoptysis, pulmonary vasculitis, and pneumonitis (48; 32.6%). Median lung ultrasound scores (interquartile range) were generally worse in posterobasal regions compared to anteroapical ones: right posterobasal 3 (0–6), left posterobasal 2.5 (0–5), right anteroapical 0 (0–3), and left anteroapical 2 (0–3). Mean PF ratio was 148 ± 71 mmHg. PF ratio was not associated with focal ARDS (Table 1), and spline analysis did not suggest non-linearity (Fig. 1, Wald test *P* = 0.612).

**Table 1:** Predictors of focal ARDS

	***N***	Focal ARDS	OR (95% CI)	***P*** value
All patients	152	16 (10.3%)	NA	NA
ARDS severity^a^				
Mild	39	4 (10.3%)^b,c^	Reference	Reference
Moderate	62	4 (6.5%)^b,c^	0.60 (0.14–2.57)	0.494
Severe	51	8 (15.7%)^b,c^	1.62 (0.45–5.86)	0.456
PF ratio < 150 mmHg	86	10 (11.6%)	1.32 (0.45–3.83)	0.614
PF ratio (mmHg)	152	16 (10.3%)	1.00 (0.99–1.01)	0.691

^a^Mild ARDS: PF ratio 201–300 mmHg and PEEP ≥ 5 cmH_2_O. Moderate ARDS: PF ratio 101–200 mmHg and PEEP ≥ 5 cmH_2_O. Severe ARDS: PF ratio ≤ 100 mmHg and PEEP ≥ 5 cmH_2_O

^b^Fisher exact test, *P* = 0.275

^c^Chi-square test for trend, *P* = 0.345

*ARDS* acute respiratory distress syndrome, *CI* confidence interval, *NA* not applicable, *OR* odds ratio, *PEEP* positive end-expiratory pressure, *PF ratio* partial pressure of oxygen in arterial blood divided by the inspired oxygen fraction

**Fig. 1:**
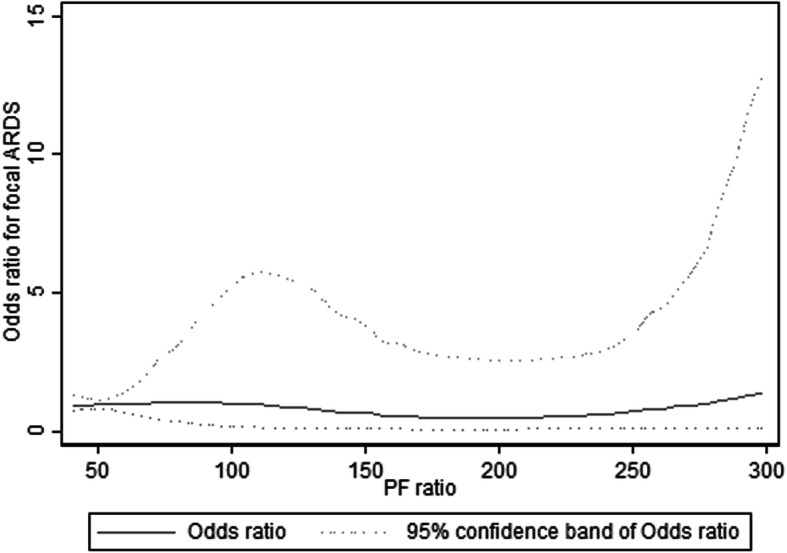
Association of odds ratio for focal ARDS with PF ratio, using a restricted cubic spline with 4 knots

We found that PF ratio was not associated with focal ARDS. A possible reason could be that the degree of oxygenation impairment is related to the *extent* of lung involvement in ARDS, rather than the *distribution* of lung involvement. To illustrate using data from our patients, for every 1 point increase in total lung ultrasound score, PF ratio decreased by 1.7 (95% CI − 3.3 to − 0.19, *P* = 0.028). In the light of our results, thoracic imaging remains a requirement for identification of focal ARDS. Admittedly, our study is limited by a single-center cohort involving medical patients and using ultrasound as the sole modality for detailed lung imaging. Our findings should therefore be validated in external cohorts, in non-medical patients, and with computed tomography.

## Data Availability

The data that support the findings of this study are available from the corresponding author, KCS, upon reasonable request.

## Abbreviations

ARDS: Acute respiratory distress syndrome
PF ratio: Ratio of partial pressure of arterial oxygen divided by fraction of inspired oxygen
